# Towards genomic-Newborn Screening: Technical feasibility of Exome Sequencing starting from dried blood spots

**DOI:** 10.1016/j.ymgmr.2024.101074

**Published:** 2024-03-20

**Authors:** Alessia Mauri, Clarissa Berardo, Davide Biganzoli, Andrea Meta, Sara Benedetti, Federica Rey, Letizia Messa, Gian Vincenzo Zuccotti, Stephana Carelli, Luisella Alberti, Cristina Cereda

**Affiliations:** a Pediatric Clinical Research Center "Romeo ed Enrica Invernizzi", Department of Biomedical and Clinical Sciences, University of Milano, 20157 Milano, Italy; b Center of Functional Genomics and Rare diseases, Department of Pediatrics, Buzzi Children's Hospital, 20154 Milano, Italy; c Department of Electronics, Information and Bioengineering (DEIB), Politecnico di Milano, 20133 Milano, Italy; d Department of Pediatrics, Buzzi Children's Hospital, 20154 Milano, Italy

**Keywords:** Genomic-NBS, Dried blood spot, NGS from DBS

## Abstract

Each year thousands of babies are born with rare genetic disorders not identified by current NBS panels, due to programs which are not yet optimal. Next-generation sequencing technologies have the potential to overcome many NBS drawbacks and provide large amounts of molecular data, broadening the number of diseases investigated. Here, we design and set up an NGS-based approach to evaluate the feasibility of NGS from dried blood spot starting from 34 DBSs.

After assessing gDNA yield and integrity, libraries were performed using three target enrichment approaches, sequenced on NS500 platform, and analyzed on commercial platform. Specifically, we focus on virtual gene panels related to highly actionable neonatal/pediatric disorders.

WES show that amount and quality of DBS-extracted gDNA are suitable for high-throughput sequencing. We obtain 500–1500 ng for each specimen, 1.7–1.8 260/280 wavelength, and DIN of 7 resulting DNA integrity, on par with traditional venous blood collection. A high read depth with 94.3% coverage uniformity is achieved for all samples.

Data results on mean coverage are comparable among the different workflows tested and demonstrate that DBS from newborn collected at birth is a suitable material for the developing of gNBS programs.

## Graphical abstract

**Figure:**
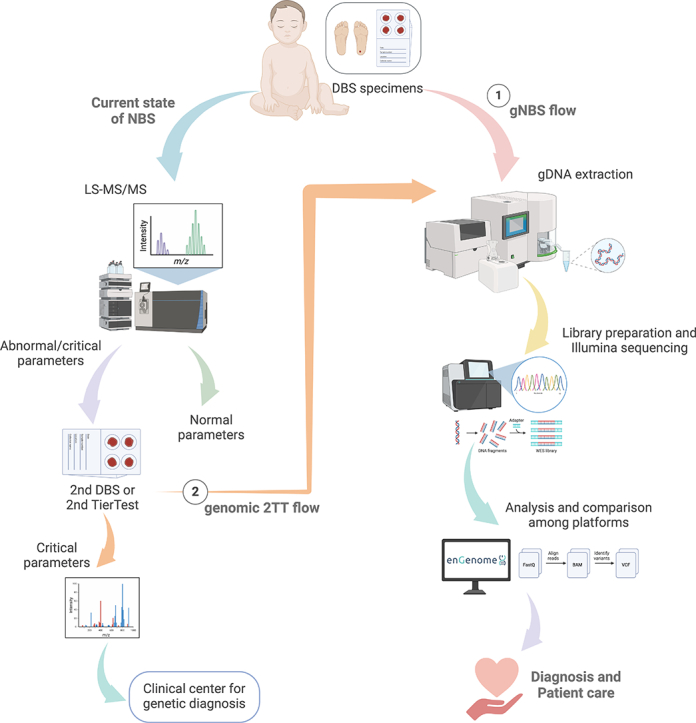

## 1 Introduction

Newborn screening (NBS) is a public health program aimed to detect neonates with rare disorders early in life, for which timely treatment or intervention is available and necessary to improve the condition [1]. To date, the more common panel of diseases screened worldwide include aminoacidemias, organic acidemias, fatty acid oxidation disorders and urea cycle defects, also known as inborn errors of metabolism (IEMs) [2]. Currently, NBS is carried out through tandem mass spectrometry (MS/MS) [3], enzyme activity evaluation [4] and polymerase chain reaction (PCR) methods on dried blood spot (DBS) specimens collected within 48–72 h of birth. Next, biochemical positives and borderline cases are tested for genetic confirmation on traditional Whole Blood collection samples (tWB), and then proceed to clinical management.

Also known as the heel prick test, DBS approach has been routine for over 50 years [5], and it presents advantages over traditional venous blood collection. Indeed, DBS is a simple, minimally invasive sampling process; it requires a small volume of blood (10–40 μl) and is stable at room temperature for extended periods of time. Moreover, DBS can be easily shipped by mail and stored [6].

Despite great success at identifying affected infants for the screened diseases, biochemical NBS program is not yet optimal and results in relatively high numbers of false positives related to environmental factors, such as dietary intake and treatment, maternal conditions, which all influence metabolites levels, thereby imposing an unnecessarily large burden on the healthcare system and parental anxiety. On the other hand, each year thousands of babies born with rare genetic disorders, of which only 10% treatable [7], that are not detected by current NBS panels [8].

Next-generation sequencing (NGS) technologies have the potential to overcome many NBS drawbacks and provide large amounts of molecular data available throughout life, broadening the diseases investigated efficiently and at minimal additional cost [9]. Recently, exploratory studies have been launched to investigate the effectiveness of using NGS in an NBS setting aimed to ensure timely diagnosis, access to treatment and better outcomes, and quality of life for infants and their families [10]^,^ [11,12].

Notably, the possibility to implement NGS as part of NBS programs requires careful evaluation of technical issues. The main challenge concerns the genomic DNA (gDNA) extraction from DBS samples suitable for NGS analyses, which may be inadequate due to degradation and the limited gDNA yield. Although it is known that source material affects coverage, and accurate and comprehensive detection of disease-causing variant, limited data are currently available on how gDNA from DBS affect the sequencing performance.

To evaluate the feasibility of Whole Exome Sequencing (WES) on DBS, and thus the potential of genomic-NBS (gNBS) as early genetic testing to reduce the number of false positives whereas increasing the diagnosis, we designed and set up an NGS-based method starting from 34 DBSs. Specifically, we achieved promising results related to features of gDNA isolated from DBS specimens collected by heel prick test and performed WES using three target enrichment methods to obtain more comprehensive insights of platforms performance. Furthermore, we compared sequencing quality control parameters and variant analyses among DBS and tWB samples, which is currently the gold standard, resulting very comparable for both matrices.

To the best our knowledge, these data demonstrate, for the first time, that DBS is a promising source material to integrate biochemical NBS and perform future gNBS programs relying on high-throughput sequencing technologies.

## 2 Materials and methods

### 2.1 Sample collection

34 anonymous DBS specimens were collected from babies at the Newborn Screening Unit, V. Buzzi Children's Hospital, Milan, using the standard heel prick procedure and stored at room temperature for gNBS up to a period of three years, while 6 peripheral blood anonymous samples were collected in EDTA tubes and stored at −20 °C until gDNA extraction.

### 2.2 gDNA extraction

Genomic DNA is extracted from DBS samples using the Chemagic™ DNA Blood Spots Kit on the Chemagic™ 360 instrument according to manufacturer's instructions (PerkinElmer, Waltham, MA, USA), with some modifications and implementation. For each specimen three punches (3.2 mm in diameter) are punched (Panthera-Puncher™ 9, PerkinElmer, MA, USA) from DBS card, corresponding to 10.30 μl [13], and placed into 1.5 ml centrifuge tubes. Red blood cell lysis buffer, proteinase K and 1 M Dithiothreitol (DTT) solution are added in appropriate volumes following standard protocol to each tube containing samples. The tubes are vortex mixed for 5 s and incubated overnight at 56 °C with agitation at 1800 rpm. After the lysis has been completed, the tubes are centrifuged briefly and the complete lysates without paper material are transferred into a deep-well-plate. The second part has been performed by robotic system based on chemagen magnetic bead technology. It is necessary to connect binding and wash buffers (containing ethanol) to the chemagic Dispenser and prefill magnetic beads and elution buffer according to the sample positions. Samples are eluted in 80/100 μl elution buffer (10 mM Tris-HCl to pH 8.0). After the DNA isolation procedure has finished, the 96-well plate is vortex mixed for 10 s and centrifuged shortly at high speed for 1 min.

DNA is purified from tWB samples using the Chemagic™ Body Fluid 200 H96 Kit on the Chemagic™ 360 instrument according to the manufacturer's protocols (PerkinElmer, Waltham, MA, USA).

The quantity and integrity of the isolated gDNA are assessed using spectrophotometry (NanoDrop One™, Thermo Fisher Scientific, MA, USA), the fluorometric assay for double-stranded DNA on Qubit Flex™ (Invitrogen, MA, USA) instrument and automated electrophoresis on Agilent 4200 TapeStation System (Agilent Technologies, CA, USA).

### 2.3 Library preparation

**Fig. 1:**
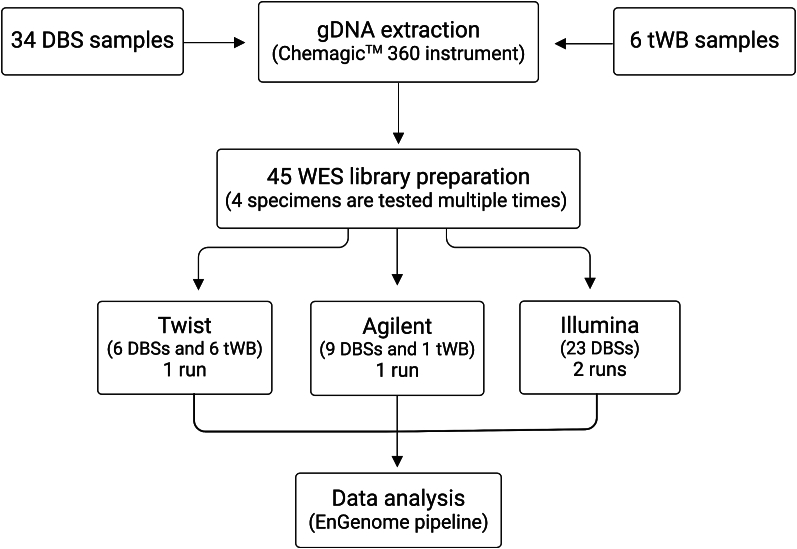
Flowchart of the WES study. (Created with BioRender.com)

WES was performed in a total of 40 samples using three commercially target enrichment kits from Twist (12 sample: 1 run), Agilent (10 samples: 1 run) and Illumina (23 samples: 2 run) companies. Notably, some DBSs are tested multiple times to compare the performance of kits for a total of 45 libraries preparation (Supplementary Table, S1). A flowchart of the WES approach is shown in Fig. 1.

12 libraries, 6 from DBS and 6 from tWB, were performed using the Twist Target Enrichment Standard Hybridization v2. Input gDNA of 100 ng was used for enzymatic DNA fragmentation and library preparation with minimal cycles [7] of PCR. The amplified indexed samples were analyzed for size using TapeStation 4200 (Agilent Technologies, CA, USA), and then pooled for the following hybridization reaction. Capture, clean-up and amplification was carried out as recommended by the manufacturer's protocol. Next, libraries from 10 DNA samples (1 tWB and 9  DBS) were carried out using the Agilent SureSelectXT Target Enrichment (V8 + NCV) System. From 40 to 100 ng were used as gDNA input for tagmentation and pre-capture PCR. Following hybridization and capture, WES libraries yield, and fragment sizes were determined using the TapeStation 4200 (Agilent Technologies, CA, USA), and then pooled. A total of 23 exomes were captured using Illumina DNA Prep with Exome 2.0 following the manufacturer's protocol and using 100 ng as input gDNA.

Overall, WES libraries were run paired end (PE) with a read length of 149 bases on Illumina NextSeq 500 (Illumina, San Diego, CA, USA) platform.

### 2.4 Data analysis

The BCL Convert on BaseSpace™ Illumina was used for FastQ generation (primary analysis). Quality control (QC) FastQC is carried out. For secondary analysis reads are aligned on the NCBI human reference genome build GRCh38 and manifest file using EnGenome eVai software (evai.engenome.com).

To assess the variation QC parameters (Total reads, Passed Filtered (PF) unique reads and Mean Coverage base depth) between the DBS and tWB groups, we employed Wilcoxon rank sum test for unpaired samples data.

### 2.5 Custom panel design

The selection of the virtual gene panel was related to IEMs [14], and conditions with high medical actionability in neonatal and pediatric age. Main criteria were: i) genes with strong association to the disease and high penetrance (>80%), ii) metabolic disorders with neonatal or pediatric onset, iii) diseases for which treatment, monitoring, and/or medical management can potentially improve clinical outcome reducing mortality/morbidity.

## 3 Results

### 3.1 Cohort study

We selected 34 anonymous DBS cards collected by heel prick procedure from infants at the Neonatal Screening Unit, V. Buzzi Children's Hospital, Milan, from 2020 to 2023. For all specimens we performed standard biochemical NBS: 6 of them were negative samples while 28 DBSs were resulted positives, hence awaiting genetic confirmation.

At the same time, we have collected 6 peripheral blood anonymous samples (tWB).

### 3.2 gDNA isolation quality control

To assess the technical suitability of WES starting from DBS, gDNA was isolated from DBS and tWB samples, achieving interesting results related to features of gDNA.

**Fig. 2:**
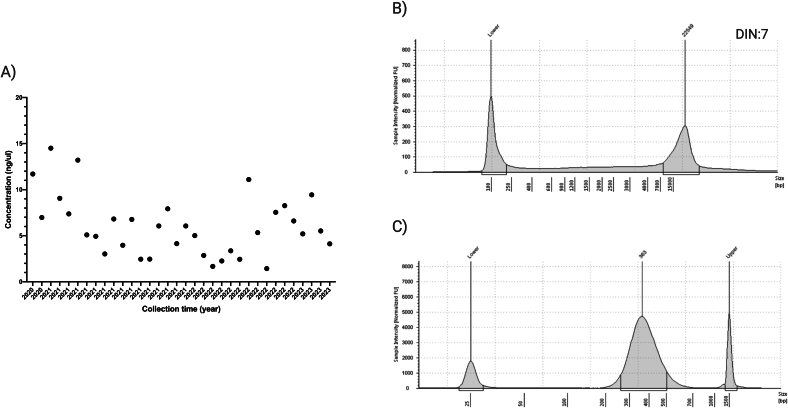
Quality assessment of gDNA and library preparation starting from representative DBS specimen. A) gDNA concentration for each DBS specimen as a function of the collection time (x-axis). B) Electropherogram of gDNA sample isolated from DBS analyzed with the Agilent Genomic DNA ScreenTape assay to determine DNA integrity by the DNA integrity number (DIN). C) Pre-capture library analysis using the Agilent D1000 ScreenTape assay. The x-axis and the y-axis represent the fragment size (bp) of gDNA and sample intensity (normalized FU), respectively.

For DBS, the gDNA concentration resulted of 200–1500 ng in 100 μl for each specimen (Table S1), while yields from 200 μl of tWB collection were tenfold higher (9000–15,000 ng in 50 μl). Noted in Fig. 2A, DBSs collected in 2020 (22G75 and 22G85), stored at room temperature and extracted three years later achieved the same gDNA yield as processed samples collected two days before by heel prick procedure. The DNA purity assessed by the 260/280 wavelength (for protein, phenol, or other contaminants) for gDNA from DBS and tWB samples showed to be in the reference values of 1.7–2 (Table S1). In addition, the integrity gDNA extracted from DBS reached a 7 DIN (DNA integrity number) ranking, comparable that of blood. Examples of gDNA integrity from isolated DBS samples, and quality assessment of libraries are displayed in Fig. 2B-C. Table 1 shows an average of the main quality parameters.

**Table 1:** Quality parameters: average of the main chemico-physical and sequencing metrics of the two sample types.

	Concentration (ng/μl)	260/280 ratio	Mean target coverage	Coverage uniformity	PCT target bases 20×
tWB	238.1	1.8	139.9	97.8	97.5
DBS	6.0	1.8	117.4	95.5	95.3

### 3.3 Sequencing quality metrics

**Fig. 3:**
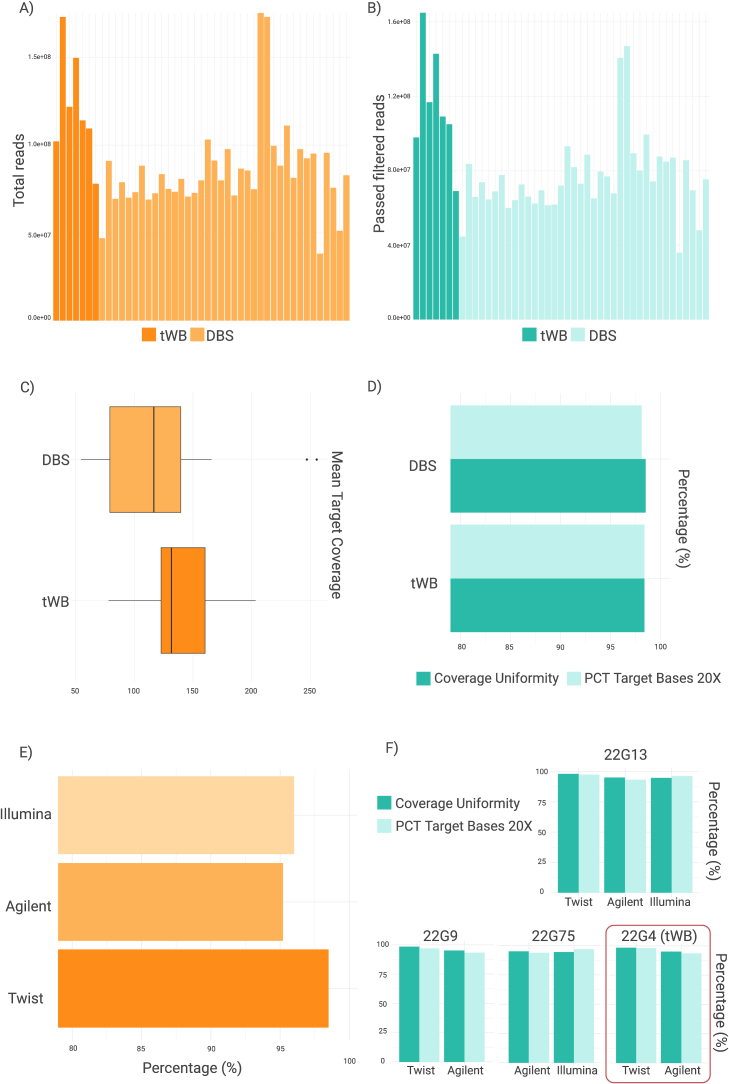
Comparison of quality metrics. A) Total reads among tWB and DBS samples, B) Number of Passed Filtered (PF) aligned reads tWB and DBS samples, C) Mean target Coverage among sample type, D) Percentage of Coverage Uniformity and of target bases with coverage above 20× among tWB and DBS samples, E) Percentage of Coverage Uniformity of three enrichment platforms, F) Percentage of Coverage Uniformity and of target bases with coverage above 20× comparable among platforms of three DBS (22G9, 22G13, 22G75) and one tWB (22G4) tested multiple times.

Libraries performed on all 40 specimens included in the study showed similar yield and size, with no significant differences in the mean base coverage depth (*p* = 0.1574) among DBS and tWB samples (Table S1). QC plots compare the total read counts and the read fractions uniquely aligned to the genome for both sample types (Fig. 3A-B). For both parameters, there is a significant difference between the two sample groups (*p* < 0.05).

The mean coverage for the 34  DBS and 6 tWB samples, reported in Fig. 3C, is in the range of 60.1 to 255.5×. Coverage uniformity achieved a mean of 95.8%, very comparable for both samples (97.8% in tWB and 95.5% in DBS), and the percentage of target bases with coverage above 20× is between 87.1% and 98.4% for both matrices (Fig. 3D).

### 3.4 Comparison among workflows

A high read depth was detected with all three-enrichment kit tested (Fig. 3E), indicating a high capture efficiency. The comparison among platforms suggests that the libraries performed with Twist workflow have improved coverage uniformity of sequencing depth than Agilent and Illumina kits, although 94.3% of coverage uniformity and 92% of target bases with coverage above 20× is achieved for all DBS samples.

To obtain a more comprehensive insight of platforms performance, three DBS (22G13, 22G9, 22G75) and one tWB (22G4) were tested multiple times. In Fig. 3F is displayed the percentage of coverage uniformity and of target bases with coverage above 20× comparable among platforms, indicating a high reproducibility of data.

### 3.5 Panel analysis

**Fig. 4:**
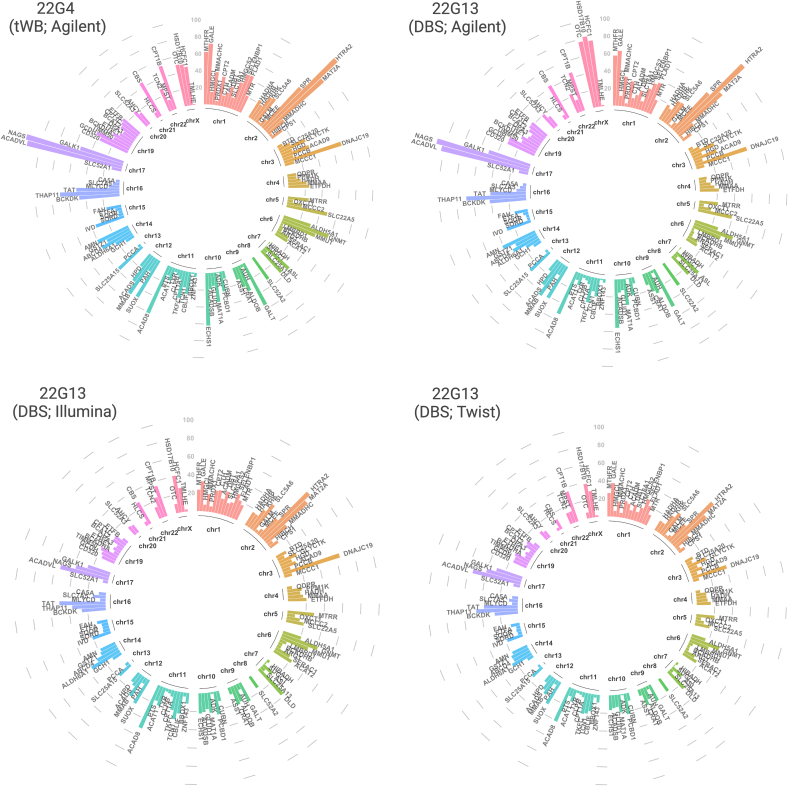
Mean coverage of each gene of the panel observed in tWB 22G4 and DBS 22G13 tested with the three workflows. (Created with ggplot2).

The panel of 128 genes associated with conditions currently included in NBS [14], mainly affecting amino acids, fatty acids, organic acids and urea cycle, and diseases with high medical actionability in neonatal and pediatric age is provided in Table S2. Fig. 4 and Table S3 show the mean coverage of each gene of panel observed in tWB 22G4 performed with Agilent workflow, and DBS 22G13 tested with all three platforms (Agilent, Illumina and Twist).

**Supplementary Fig 1:**
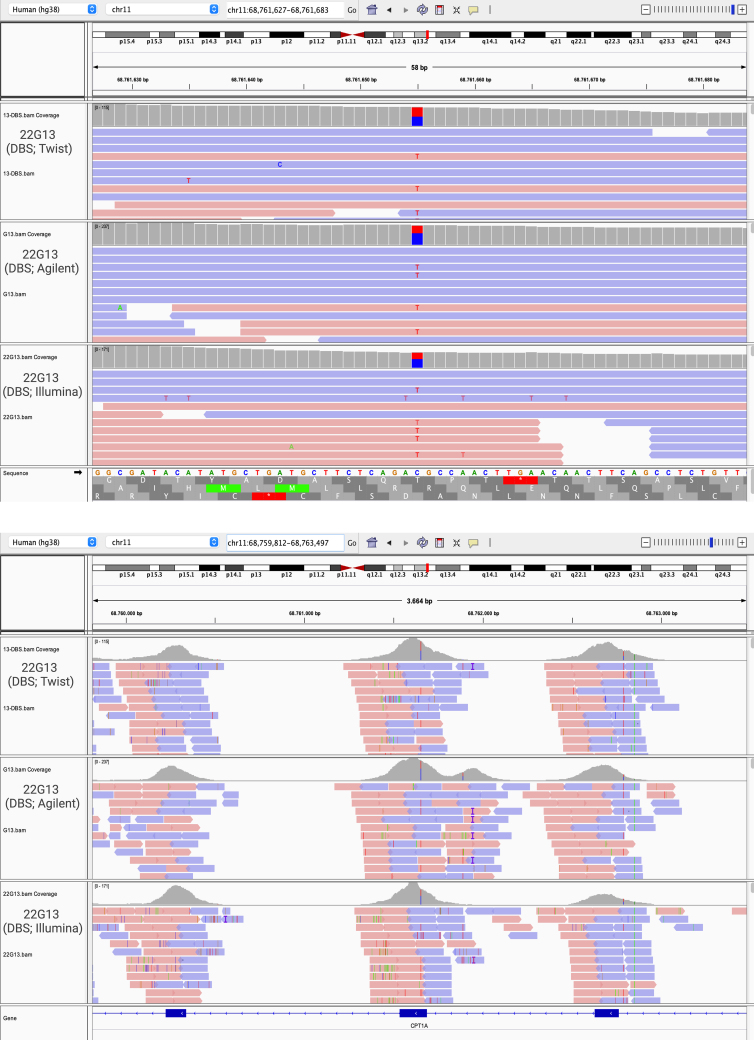
Visualization in IGV of the polymorphism (rs111407620) in three datasets of the 22G13 sample.

Due to the mean coverage <10×, we explored the *CPT1A* gene in more detail. Fig. S1 illustrates the identified polymorphism rs111407620 (NM_001876.4:c.1908G>A) in three datasets of the 22G13, proving kit reliability.

Out of a total of 34 samples evaluated in WES starting from DBS, 28 are selected and analyzed on enGenome's eVai software, focusing on gene panel. The identified variants in these DBS samples are compared to those previously detected on tWB ones, obtaining 100% of concordance among matrices for IEMs.

## 4 Discussion

The possibility to implement genomic sequencing in NBS programs has great relevance for timely diagnosis, access to treatment and medical management that can potentially improve clinical outcomes and reduce mortality/morbidity. Besides the advantages, NGS in newborns requires the evaluation of ethical, economic and feasibility issues. The first two aspects have already been discussed extensively elsewhere [15]^,^ [16], but the feasibility aspect, including technical challenges, experimental times and test specificity, needs to be addressed in more details. Here, we focused on the technical insight, specifically related to gDNA isolation and sequencing from DBS samples.

Although it is known that the source of material affects both coverage and accurate detection of disease-causing variant, limited data are currently available on how gDNA from DBS affects the sequencing performance [17,18]. To date, only two studies were performed on the gDNA extraction methods from DBS, and they did not provide details on technological features such as total reads counts, mean coverage and coverage uniformity. One of these reports that casual variants for inherited metabolic diseases can be detected starting from DBS-extracted gDNA [17]. In the latter, Mortensen et al. were able to carry out WES using DBS obtained from peripheral blood samples collected in EDTA tubes and then spotted onto cards [18].

Therefore, given the minimally invasive sampling by heel prick and the direct collection on DBS, the easy transport and storage, and the cost containment compared to conventional EDTA tubes for blood collection [19], further implementations of the DBS workflow were needed to explore the full potential of DBS sampling as a source for genomic analysis. To this end, we developed a WES-based method starting from 34  DBSs of newborn collected at birth compared with 6 tWB specimens.

Specifically, we achieved interesting findings related to the features of gDNA isolated from DBSs. Although the gDNA quantity from 3 punches of DBS (about 10 μl) was one-tenth of the yield of gDNA obtained from 200 μl of tWB, the quality from both matrices was adequate for downstream library preparation. It is important to highlight that samples collected three years before and stored at room temperature achieved the same quality as processed samples collected two days before by heel prick method. Therefore, this suggests that the variability in gDNA yield that we observed in our study should be not due to storage time, but most likely to the quality of the drop during collection procedure. This aspect will be further investigated in the near future to standardize the blood amount in single spot. Moreover, yield and profiles of WES libraries, indeed, showed close to identical performance in both sample types. Also, mean coverage and total number of SNVs and INDELs were similar in both source materials. The SNV Het/Hom zygosity ratio of 9:1 is resulted comparable for both matrices, according to the ACMG guidelines to prevent the false-positive homozygous segments. Rationally, a criticism of this approach should be the absence of the possibility of being able to compare the same blood sample with the two collection methods. But in this specific case, we were dealing with blood samples from newborns just a few hours old from whom, unless for medical necessity, it was unethical to take further blood sample to evaluate the collection in the EDTA tube.

The comparison among platforms highlighted a high read depth among all three tested kits, indicating a high capture efficiency. Moreover, three DBS (22G13, 22G9, 22G75) and one tWB (22G4) were tested multiple time to obtain a more comprehensive insight of kits performance and importantly the obtained results indicate a high reproducibility of observations. In addition, the three datasets of 22G13 show concordance in the identification of variants even when mean coverage results <10× (such as *CPT1A* gene), demonstrating the reliability of DBS and platforms tested.

Variant interpretation analysis was performed on 28 exomes on DBS specifically issued from patients with well-established IEM. When we compared observed variants to those previously detected on tWB, we obtained 100% of concordance among matrices.

In this context, our findings demonstrate that DBS is a promising source material for future gNBS programs relying on high-throughput sequencing technologies. Specifically, data results comparable among the different platforms, and furthermore, between the two matrices tested. This high reproducibility of data shows the possibility of quick diagnosis, specifically for those diseases where early diagnosis, treatment or intervention are essential to improve the condition of the affected patient.

The following are the supplementary data related to this article.

Supplementary material 1 Quality metrics. (*) recalls tWB samples, in bold are reported samples tested multiple times. Supplementary material 1

Supplementary material 2 Virtual gene panel associated with conditions included in NBS program. Supplementary material 2

Supplementary material 3 Mean coverage of each gene of the panel observed in tWB 22G4 and DBS 22G13 tested with the three workflow. Supplementary material 3

## Funding

Better rEal-world healTh-daTa distributEd analytics Research platform (BETTER 101136262); call: HORIZON-HLTH-2023-TOOL-05.

## CRediT authorship contribution statement

**Alessia Mauri:** Writing – original draft, Methodology, Investigation, Formal analysis, Data curation, Conceptualization. **Clarissa Berardo:** Writing – review & editing, Data curation. **Davide Biganzoli:** Formal analysis. **Andrea Meta:** Methodology. **Sara Benedetti:** Methodology, Formal analysis. **Federica Rey:** Writing – review & editing. **Letizia Messa:** Writing – review & editing. **Gian Vincenzo Zuccotti:** Writing – review & editing. **Stephana Carelli:** Writing – review & editing. **Luisella Alberti:** Data curation. **Cristina Cereda:** Writing – review & editing, Supervision, Conceptualization.

## Declaration of competing interest

The authors declare no conflicts of interest.

## Data Availability

All FASTQ files will be made available on request.
