# Supplementary material for: Towards genomic-Newborn Screening: Technical feasibility of Exome Sequencing starting from dried blood spots
Source: Mol Genet Metab Rep. 2024 Mar 20;39:101074. doi: 10.1016/j.ymgmr.2024.101074 (PMC10966309; doi:10.1016/j.ymgmr.2024.101074)
Supplement: Supplementary material 1 — Quality metrics. (*) recalls tWB samples, in bold are reported samples tested multiple times. [file mmc2.docx]

|  |  | |  |  | |  |  |  |  |  |  |  |  |  |
| --- | --- | --- | --- | --- | --- | --- | --- | --- | --- | --- | --- | --- | --- | --- |
|  | | ID | Collection time (year) | | Concentration (ng/uL) | 260/280 ratio | Total Reads | | PF reads not duplicated | On-target bases (%) | Mean Target Coverage | Coverage  Uniformity (%) | SNV | INDEL |
| Twist | | 22G2* | 2022 | | 175 | 1.8 | 102,236,760 | | 97,884,502 | 29.7 | 117.6 | 98.3 | 116,546 | 23,684 |
|  |  | 22G3* | 2022 | | 308 | 1.7 | 172,910,448 | | 164,875,870 | 30.0 | 203.5 | 98.4 | 118,667 | 27,047 |
|  |  | **22G4*** | 2022 | | 215 | 2.1 | 121,914,842 | | 116,896,551 | 30.1 | 144.6 | 98.4 | 136,187 | 27,707 |
|  |  | 22G5* | 2022 | | 200 | 1.8 | 149,763,882 | | 142,750,187 | 29.9 | 176.1 | 98.3 | 116,194 | 25,631 |
|  |  | 22G6* | 2022 | | 314 | 1.9 | 114,022,708 | | 109,121,626 | 29.6 | 131.7 | 98.3 | 117,416 | 24,631 |
|  |  | 22G7* | 2022 | | 240 | 1.7 | 109,535,170 | | 105,079,663 | 30.0 | 128.1 | 98.4 | 118,189 | 24,974 |
|  |  | **22G9** | 2022 | | 5.01 | 1.9 | 46,959,856 | | 44,728,062 | 29.6 | 54.5 | 98.4 | 113,968 | 21,107 |
|  |  | 22G10 | 2022 | | 2.86 | 1.7 | 91,055,220 | | 83,628,339 | 28.1 | 99.8 | 98.5 | 118,439 | 24,160 |
|  |  | 22G11 | 2022 | | 1.67 | 1.8 | 69,378,916 | | 66,009,754 | 29.7 | 81.1 | 98.4 | 117,665 | 23,329 |
|  |  | 22G12 | 2022 | | 2.26 | 1.8 | 78,804,504 | | 73,852,858 | 29.2 | 91.0 | 98.3 | 119,314 | 24,759 |
|  |  | **22G13** | 2022 | | 3.36 | 2.1 | 69,986,860 | | 64,475,838 | 28.2 | 77.2 | 98.3 | 140,617 | 26,296 |
|  |  | 22G14 | 2022 | | 2.44 | 1.7 | 73,180,032 | | 68,804,127 | 29.3 | 84.1 | 98.4 | 122,535 | 24,041 |
| Agilent | | 22G71 | 2021 | | 14.50 | 1.7 | 88,377,182 | | 77,693,163 | 31.8 | 85.2 | 94.8 | 118,065 | 48,067 |
|  |  | 22G72 | 2021 | | 9.06 | 1.7 | 68,941,534 | | 60,105,685 | 33.4 | 69.6 | 94.6 | 107,849 | 39,851 |
|  |  | 22G74 | 2021 | | 7.37 | 1.8 | 72,506,178 | | 64,084,175 | 33.6 | 73.7 | 95.2 | 105,770 | 39,922 |
|  |  | **22G75** | 2020 | | 11.70 | 1.6 | 83,508,606 | | 72,666,447 | 33.2 | 84.0 | 95.1 | 115,778 | 42,592 |
|  |  | 22G76 | 2021 | | 13.20 | 1.8 | 75,005,554 | | 66,194,299 | 34.6 | 78.6 | 94.7 | 112,556 | 42,679 |
|  |  | 22G79 | 2022 | | 11.10 | 1.6 | 73,342,186 | | 62,431,955 | 30.2 | 67.0 | 94.9 | 103,634 | 42,861 |
|  |  | 22G80 | 2022 | | 5.33 | 1.9 | 80,821,080 | | 69,521,612 | 33.5 | 81.9 | 95.1 | 117,800 | 41,910 |
|  |  | **22G9** | 2022 | | 5.01 | 1.9 | 70,728,732 | | 61,432,687 | 32.4 | 69.3 | 95.1 | 110,579 | 38,187 |
|  |  | **22G4*** | 2022 | | 215 | 2.1 | 78,056,472 | | 69,156,555 | 33.1 | 78.1 | 95.1 | 124,644 | 45,280 |
|  |  | **22G13** | 2022 | | 3.36 | 2.1 | 72,760,968 | | 61,715,138 | 31.7 | 69.8 | 95.1 | 131,682 | 39,928 |
| Illumina | | **22G13** | 2022 | | 3.36 | 2.1 | 79,918,232 | | 72,064,197 | 76.5 | 123.7 | 95.0 | 147,937 | 22,694 |
|  |  | 22G83 | 2021 | | 5.09 | 1.8 | 103,093,410 | | 93,140,204 | 71.4 | 151.9 | 95.2 | 127,059 | 20,576 |
|  |  | 22G84 | 2021 | | 4.93 | 1.7 | 91,198,128 | | 81,855,155 | 76.6 | 143.6 | 94.9 | 123,957 | 19,651 |
|  |  | 22G85 | 2020 | | 6.98 | 1.7 | 79,836,464 | | 73,080,672 | 72.8 | 122.0 | 95.1 | 126,580 | 20,120 |
|  |  | 22G86 | 2021 | | 3.02 | 1.6 | 97,792,266 | | 88,540,034 | 74.4 | 147.9 | 95.5 | 127,649 | 20,795 |
|  |  | 22G87 | 2021 | | 6.81 | 1.8 | 71,278,020 | | 65,119,658 | 76.6 | 113.9 | 94.8 | 143,280 | 21,679 |
|  |  | 22G88 | 2021 | | 3.96 | 1.6 | 86,684,870 | | 79,473,986 | 68.8 | 124.5 | 95.2 | 125,035 | 19,602 |
|  |  | 22G89 | 2021 | | 6.77 | 1.9 | 85,682,378 | | 77,010,067 | 77.5 | 136.4 | 94.8 | 123,408 | 19,385 |
|  |  | 22G90 | 2021 | | 2.45 | 1.7 | 74,815,610 | | 67,900,965 | 76.9 | 119.4 | 94.8 | 121,339 | 19,047 |
|  |  | 22G92 | 2022 | | 1.43 | 1.7 | 175,211,328 | | 140,671,074 | 77.0 | 247.1 | 96.0 | 128,145 | 21,071 |
|  |  | 22G94 | 2021 | | 2.46 | 1.9 | 172,913,932 | | 146,898,426 | 77.1 | 255.5 | 95.4 | 131,042 | 21,460 |
|  |  | **22G75** | 2020 | | 11.70 | 1.6 | 99,709,188 | | 89,456,505 | 76.7 | 155.4 | 94.4 | 125,728 | 20,283 |
|  |  | 22G77 | 2021 | | 6.06 | 2.1 | 88,321,314 | | 80,190,920 | 76.3 | 135.0 | 94.3 | 126,283 | 20,089 |
|  |  | 22G78 | 2022 | | 7.54 | 1.8 | 111,093,956 | | 99,515,648 | 73.0 | 165.9 | 95.3 | 130,452 | 21,412 |
|  |  | 23G15 | 2021 | | 7.92 | 2.1 | 81,290,372 | | 74,162,430 | 75.5 | 127.0 | 94.8 | 124,737 | 19,665 |
|  |  | 23G16 | 2021 | | 4.14 | 2,1 | 97,705,774 | | 87,602,949 | 76.1 | 149.0 | 94.7 | 128,108 | 20,657 |
|  |  | 23G17 | 2023 | | 5.20 | 1,8 | 92,518,470 | | 84,812,016 | 71.0 | 136.8 | 95.5 | 129,575 | 21,407 |
|  |  | 23G18 | 2022 | | 8.26 | 1,8 | 95,312,618 | | 87,018,086 | 71.0 | 140.6 | 95.1 | 129,138 | 21,185 |
|  |  | 23G19 | 2023 | | 9.44 | 1,7 | 38,183,500 | | 35,977,147 | 74.0 | 60.1 | 94.6 | 131,157 | 19,402 |
|  |  | 23G20 | 2022 | | 6.60 | 1,6 | 95,661,884 | | 85,623,357 | 75.9 | 147.5 | 94.5 | 124,459 | 19,849 |
|  |  | 23G21 | 2023 | | 5.52 | 1,9 | 75,727,210 | | 69,483,570 | 73.1 | 114.0 | 94.9 | 126,663 | 20,195 |
|  |  | 23G22 | 2021 | | 6.06 | 1,9 | 51,249,984 | | 48,080,401 | 71.2 | 76.6 | 94.3 | 125,051 | 19,647 |
|  |  | 23G23 | 2023 | | 4.12 | 2,1 | 82,814,288 | | 75,347,361 | 76.4 | 129.6 | 94.8 | 122,922 | 19,556 |

Table S1: Quality metrics. (*) recalls tWB samples, in bold are reported samples tested multiple times.
