# Supplementary material for: Towards genomic-Newborn Screening: Technical feasibility of Exome Sequencing starting from dried blood spots
Source: Mol Genet Metab Rep. 2024 Mar 20;39:101074. doi: 10.1016/j.ymgmr.2024.101074 (PMC10966309; doi:10.1016/j.ymgmr.2024.101074)
Supplement: Supplementary material 2 — Virtual gene panel associated with conditions included in NBS program. [file mmc3.docx]

| \| ABCD4 \| \| --- \| \| ACAD8 \| \| ACAD9 \| \| ACADM \| \| ACADS \| \| ACADSB \| \| ACADVL \| \| ACAT1 \| \| ACAT2 \| \| ADK \| \| AHCY \| \| ALDH5A1 \| \| ALDH6A1 \| \| ALDOB \| \| AMN \| \| ARG1 \| \| ASL \| \| ASS1 \| \| AUH \| \| BBOX1 \| \| BCAT2 \| \| BCKDHA \| \| BCKDHB \| \| BCKDK \| \| BTD \| \| CA5A \| \| CBLIF \| \| CBS \| \| CD320 \| \| CLPB \| \| CPS1 \| \| CPT1A \| \| CPT1B \| \| CPT1C \| \| CPT2 \| \| CRAT \| \| CTH \| \| CUBN \| \| DBT \| \| DLD \| \| DNAJC19 \| \| ECHS1 \| \| ETFA \| \| ETFB \| \| ETFDH \| \| ETHE1 \| \| FAH \| \| FLAD1 \| \| GALE \| \| GALK1 \| \| GALM \| \| GALT \| \| GCDH \| \| GCH1 \| \| GLUD1 \| \| GLYCTK \| \| GNMT \| \| GSTZ1 \| \| HADH \| \| HADHA \| \| HADHB \| \| HCFC1 \| \| HGD \| \| HIBADH \| \| HIBCH \| \| HLCS \| \| HMGCL \| \| HMGCS2 \| \| HPD \| \| HSD17B10 \| \| HTRA2 \| \| IVD \| \| KHK \| \| LMBRD1 \| \| MAT1A \| \| MAT2A \| \| MCCC1 \| \| MCCC2 \| \| MCEE \| \| MLYCD \| \| MMAA \| \| MMAB \| \| MMACHC \| \| MMADHC \| \| MMUT \| \| MPST \| \| MTHFR \| \| MTR \| \| MTRR \| \| NAGS \| \| OPA3 \| \| OTC \| \| OXCT1 \| \| PAH \| \| PCBD1 \| \| PCCA \| \| PCCB \| \| PPM1K \| \| PRDX1 \| \| PTS \| \| QDPR \| \| SELENBP1 \| \| SERAC1 \| \| SLC16A1 \| \| SLC22A5 \| \| SLC25A13 \| \| SLC25A15 \| \| SLC25A20 \| \| SLC52A1 \| \| SLC52A2 \| \| SLC52A3 \| \| SLC5A6 \| \| SLC7A5 \| \| SORD \| \| SPR \| \| SQOR \| \| SUGCT \| \| SUOX \| \| TAT \| \| TAZ \| \| TCN1 \| \| TCN2 \| \| THAP11 \| \| TIMM50 \| \| TKFC \| \| TMLHE \| \| TYR \| \| ZNF143 \| |
| --- | --- | --- | --- | --- | --- | --- | --- | --- | --- | --- | --- | --- | --- | --- | --- | --- | --- | --- | --- | --- | --- | --- | --- | --- | --- | --- | --- | --- | --- | --- | --- | --- | --- | --- | --- | --- | --- | --- | --- | --- | --- | --- | --- | --- | --- | --- | --- | --- | --- | --- | --- | --- | --- | --- | --- | --- | --- | --- | --- | --- | --- | --- | --- | --- | --- | --- | --- | --- | --- | --- | --- | --- | --- | --- | --- | --- | --- | --- | --- | --- | --- | --- | --- | --- | --- | --- | --- | --- | --- | --- | --- | --- | --- | --- | --- | --- | --- | --- | --- | --- | --- | --- | --- | --- | --- | --- | --- | --- | --- | --- | --- | --- | --- | --- | --- | --- | --- | --- | --- | --- | --- | --- | --- | --- | --- | --- | --- | --- |

Table S2: Virtual gene panel associated with conditions included in NBS program.
